# Understanding Medical Students’ Perceptions of Failure in Medical School

**DOI:** 10.7759/cureus.74024

**Published:** 2024-11-19

**Authors:** Mubeen Toufiq, Abdullah Ahmed, Khadijah Ahmed, Nabeeha Toufiq, Umer Rajaratnam, Shimul Williams, Eyad Jamileh, Munir Ahmed

**Affiliations:** 1 Respiratory Medicine, Princess Alexandra Hospital, London, GBR; 2 Family Medicine, Whipps Cross Hospital, London, GBR; 3 Gastroenterology, University of Leeds, Leeds, GBR; 4 Paediatrics, King's College London, London, GBR; 5 Trauma and Orthopaedics, Sandwell General Hospital, London, GBR; 6 Pathology, Princess Alexandra Hospital, London, GBR; 7 Gastroenterology, Royal Blackburn Hospital, Blackburn, GBR; 8 Physics, Leyton Sixth Form College, London, GBR

**Keywords:** exams, failure, learning, medical school, student

## Abstract

This paper explores medical students' perceptions of failure through a qualitative approach, using semi-structured interviews to gather insights from six students across different academic years at Queen Mary University of London. The study aims to understand how students define failure, its causes, and its impact on their academic and personal lives. Key findings reveal that failure is perceived as multifaceted, influenced by internal and external expectations, and evolves throughout medical school. The impact of failure is significant, affecting students' motivation, mental health, and coping mechanisms. While students sought both formal and informal support, barriers such as stigma and a lack of awareness hindered access to help. The study concludes that fostering an open dialogue on failure and integrating support systems could improve students' experiences, better preparing them for the uncertainties of clinical practice. Limitations include the small sample size and focus on a single institution. Further research is suggested to broaden the understanding of failure at different stages of medical education.

## Introduction

Failure is an inevitable part of medical practice, yet medical students often enter their training with little to no prior experience in dealing with academic failure [1]. Many students progress through their pre-clinical years, which emphasize certainty and correct answers in exams, without encountering significant academic setbacks [2]. However, clinical practice presents a different reality, characterized by uncertainties and occasional failures, such as prescribing errors or adverse patient outcomes [3, 4]. The lack of preparedness in handling these failures may contribute to the stigma surrounding failure and reluctance to seek support, a phenomenon that has been linked to high rates of burnout among medical professionals with estimates ranging from 25% to 60% [5]. The impact of failure extends beyond burnout, affecting students academically, mentally, socially, and financially with potential repercussions for patient care [5-7]. Evidence suggests that students who struggle with failure during medical school are more likely to encounter difficulties in their professional practice, underscoring the importance of early intervention [8].

Despite the critical nature of this issue, the existing literature on medical students' perceptions of failure remains limited. Responses from students on this topic often lack depth, making it challenging to grasp the full complexity of their experiences [9]. Understanding how medical students perceive failure is crucial, as their views shape how they engage with available support systems. By fostering a deeper comprehension of these perceptions, we can better support students in developing effective coping mechanisms for failure, which may in turn inform policy changes aimed at reducing the stigma and improving student outcomes.

This study aims to explore medical students' perceptions of failure to increase awareness and provide insight into how students view and manage failure during their training. This research may potentially influence how failure is addressed in medical education and inform strategies to better support students throughout their academic journey.

## Materials and methods

Literature review

Before starting the study, a literature review was conducted to explore students’ perceptions of failure in medical school. This was to provide context and background by identifying current literature and common themes on failure in medical school from a student perspective. It was used as a tool to identify strengths and weaknesses in the current literature, to identify any gaps in the literature, and to help inform the focus of this research study.

Two databases were used, PubMed and Education Research Complete, with the initial search being on PubMed. The inclusion criteria for this literature search were based on articles that explored the perceptions of failure of medical students and support mechanisms to help students with failure.

**Figure 1:**
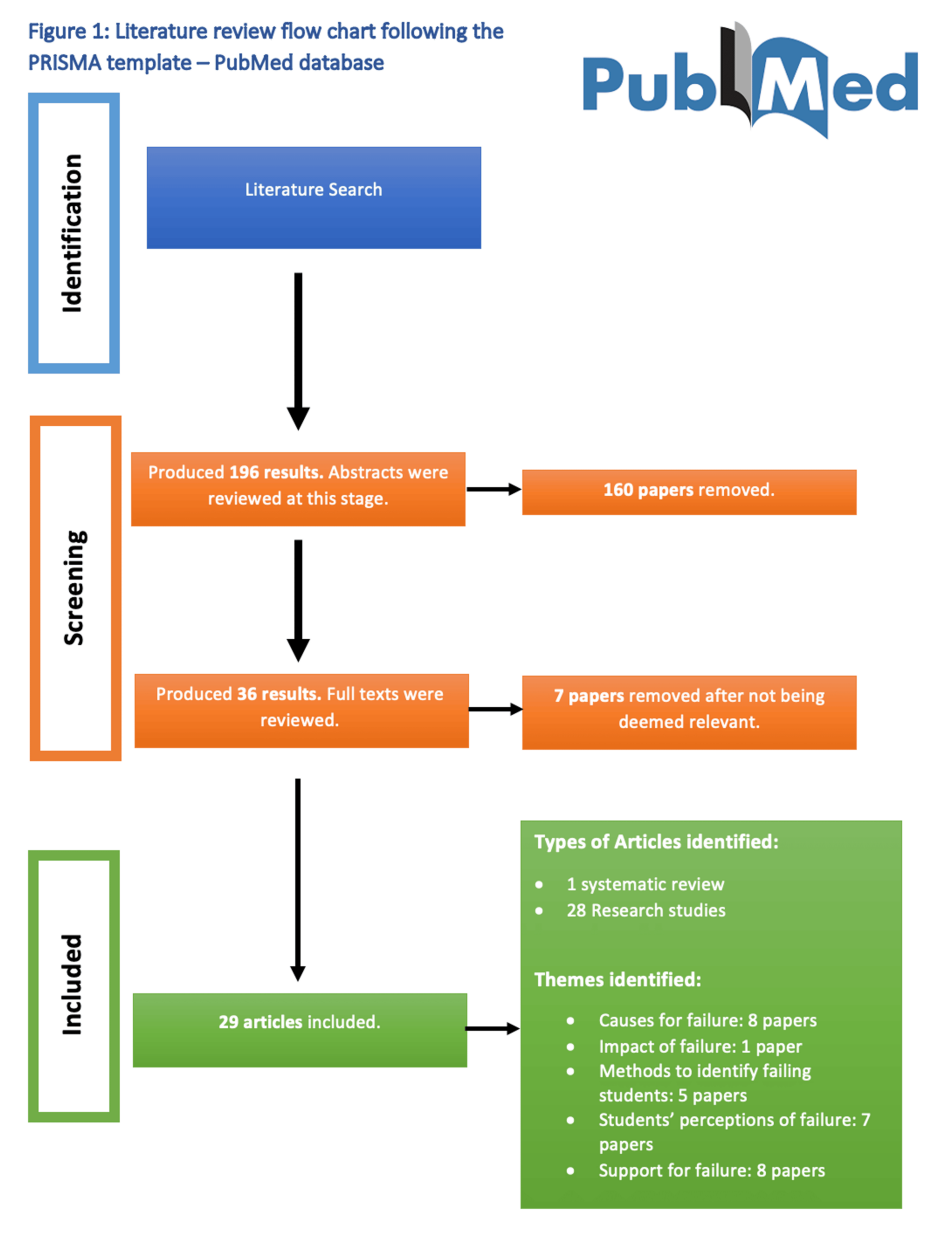
Literature review flow chart following the PRISMA template for PubMed database

A hundred and ninety-six results were produced in the initial search of the PubMed database. These research papers then had their abstracts reviewed and 36 research papers were deemed relevant. These articles had their full texts analyzed and a further seven papers were removed as they were classed as irrelevant to the literature search. This resulted in 29 papers being identified as relevant and used in the final literature review. Figure 1 which is a PRISMA flow chart summarises the literature review process for the PubMed database. 

**Figure 2:**
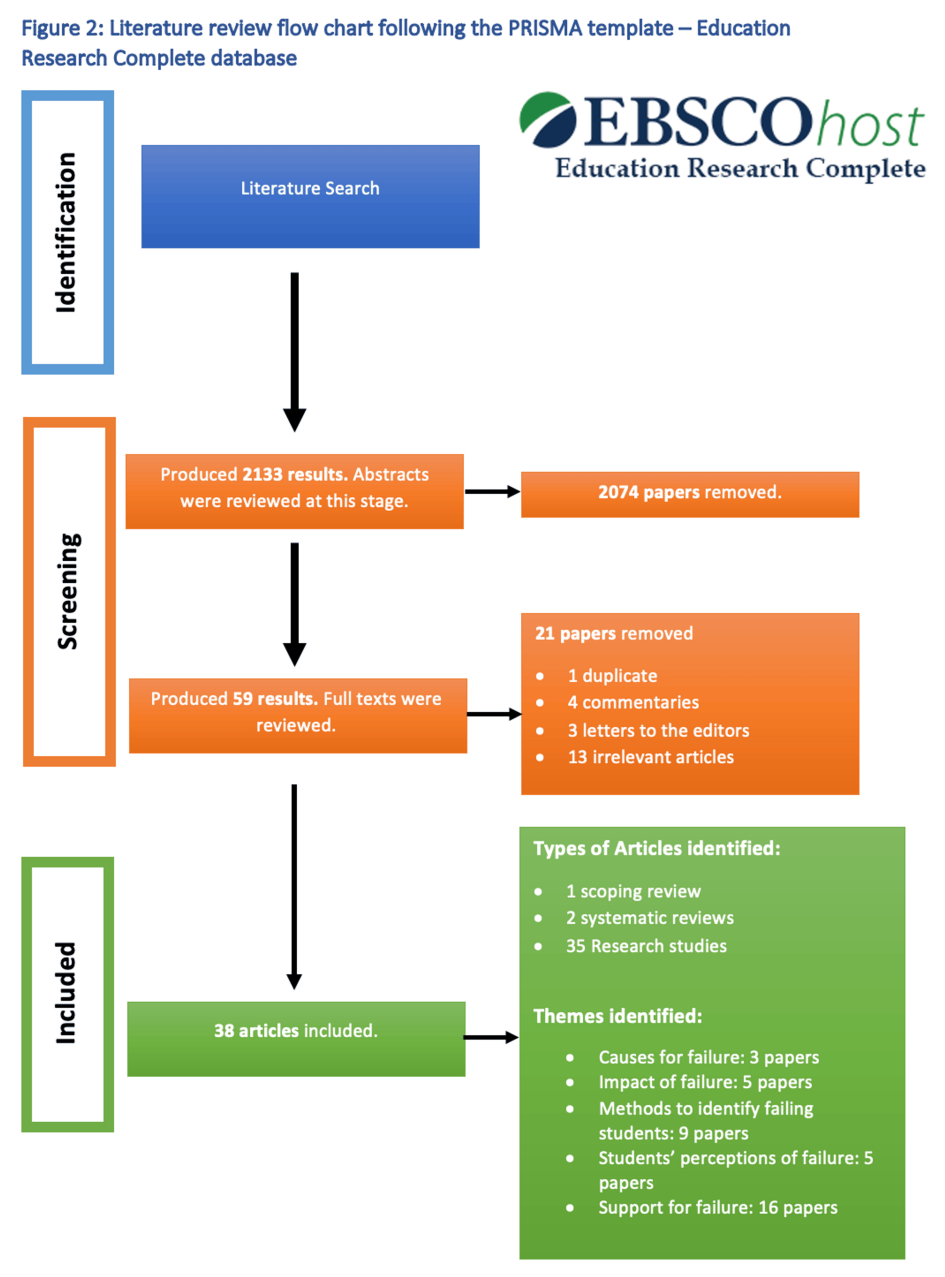
Literature review flow chart following the PRISMA template for Education Research Complete database

**Figure 3:**
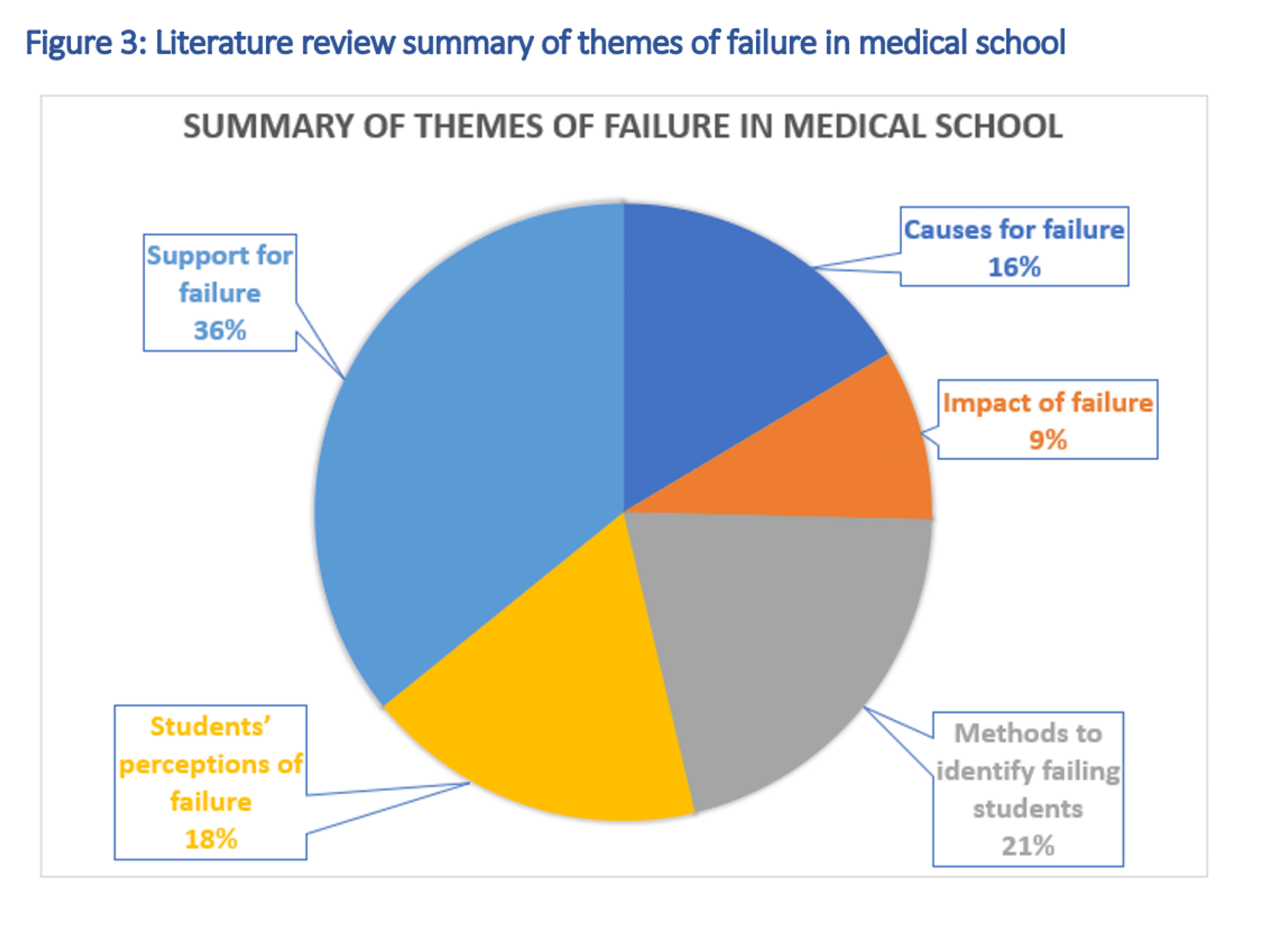
Literature review summary of themes of failure in medical school

Two thousand hundred and thirty-three results were produced in the initial search of the Education Research Complete database. These research papers then had their abstracts reviewed and 59 research papers were deemed relevant. These articles had their full texts analysed and a further 21 papers were removed. This resulted in 38 papers being identified as relevant and used in the final literature review. Figure 2 which is a PRISMA flow chart summarises the literature review process for the Education Research Complete database. Figure 3 summarises the themes found in both the PubMed and Education Research Complete databases.

Six major themes were identified, with support mechanisms for students being the most prominent [10-15]. However, only 18% of the literature directly reflected students' perspectives on failure. The majority of studies employed qualitative research methods, primarily semi-structured interviews, which provided flexibility and allowed participants to comfortably share their experiences [10, 15]. While mixed-methods approaches also offered valuable insights, the sensitive nature of the topic often led to more candid responses in one-on-one interviews rather than focus groups [13]. Despite some studies incorporating multiple themes, there is a need for more comprehensive research that encompasses the full range of students' experiences with failure. Furthermore, the limited data on how perceptions of failure evolve throughout medical school suggests that further exploration is necessary to gain a holistic understanding of this complex issue [16].

Methodology of study 

This study aims to address the gaps in the literature review by investigating the causes, impact, and support mechanisms related to failure while examining how perceptions of failure change over time. By doing so, this study will provide a more complete understanding of medical students' experiences with failure, contributing to the existing literature and offering practical recommendations for medical education.

This study employed a qualitative methodology, specifically semi-structured interviews, to explore medical students' perspectives on academic failure. A qualitative approach with an interpretivist lens was chosen as it effectively captures subjective opinions central to this topic. Quantitative methods were deemed unsuitable as they risk oversimplifying nuanced views and opinions, which are difficult to quantify [17].

Semi-structured interviews were selected for their flexibility, allowing participants to elaborate on their experiences, which is critical for an in-depth understanding of failure. A structured topic guide, based on themes identified in the literature review (e.g., causes, impacts, support, and evolving perceptions of failure), directed the interviews. Although semi-structured interviews provide rich data, they are dependent on the interviewer’s skills in facilitation, adaptability, and rapport-building [17]. A pilot interview was conducted to refine the topic guide and interview technique based on feedback [18].

Limitations of this approach include the time and effort required to conduct interviews, necessitating a smaller sample size compared to other methods like questionnaires [17]. However, interviews provide higher-quality responses, especially on sensitive topics such as failure in medical school. Due to time constraints and limited resources, a mixed-methods approach was not feasible, and only one researcher conducted and analyzed the interviews, limiting researcher triangulation [19]. Focus groups were avoided, as participants may feel restricted discussing personal experiences in a group setting [20].

Data were analyzed using thematic analysis, following Braun and Clarke’s emergent coding model [21]. This method was chosen for its flexibility in identifying and organizing themes within the data. A purposive sampling strategy was employed to include students from each year of medical school, enhancing participant triangulation and allowing for an exploration of how perceptions of failure evolve over time. The sample was limited to one student per year due to time and resource constraints, and a convenience sampling approach was used, which may introduce bias and limit the generalisability of the findings [17].

Breakdown of methodology

Inclusion Criteria

Medical students enrolled in the A100 and A101 programs at the Faculty of Medicine and Dentistry, Queen Mary University of London (QMUL), were eligible to participate in the study. All medical students from Years one to five, including those pursuing an intercalated degree, were considered potential participants.

Exclusion Criteria

Students who were not enrolled in the A100 or A101 Medicine programs at Queen Mary University of London (QMUL) during the study period were excluded from participation. Approval was obtained from a gatekeeper, specifically the head of the student support team for the Faculty of Medicine and Dentistry, to contact and recruit medical students at QMUL. Potential participants who met the selection criteria received recruitment notices via email through the student association bulletin.

Interviews

This study utilized semi-structured interviews conducted on Microsoft Teams, chosen for its integration with the university’s email system. Six medical students participated after expressing interest via email. Following feedback from a pilot interview, the interview process was refined to reduce the length of the initial briefing, clarify the line of questioning, and incorporate more probing questions. Interview questions were shared with participants through Microsoft Teams chat to enhance the flow of the conversation. Please see Appendix 1 for the semi-structured interview questions used.

The interviews, lasting 45-60 minutes, took place between the researcher and each participant. Each interview began with a briefing on the study's purpose, a review of the participant information sheet, and a reminder that participants could withdraw from the study or seek emotional support if necessary. Informed consent was confirmed before the interviews commenced. The interview explored students' perceptions of failure in medical school, focusing on changes in their views over time, the positive and negative impacts of failure, and any experiences supporting peers through similar challenges. The session concluded with a summary and an opportunity for participants to ask additional questions.

The interviews were recorded on Microsoft Teams, which generated automatic transcripts. These transcripts were anonymized to remove identifying information and were checked for accuracy by the researcher. Once anonymized transcripts were prepared, all recordings and non-anonymized data were deleted. The anonymized transcripts were securely stored on encrypted devices, with only the researcher and research supervisors having access. Data management adhered to the General Data Protection Regulation (GDPR) and Queen Mary University of London’s Research Data Access and Management Policy.

Thematic analysis followed the six-step process described by Braun and Clarke, using an emergent coding framework (21). Initially, transcripts were reviewed without pre-established codes. Codes, defined as elements of the data that were of interest, were then systematically identified and grouped. This rigorous process ensured that each piece of data was given equal consideration. Similar codes were then organized into broader themes, which were further refined into key themes representing meaningful patterns within the data [17]. The study’s findings will explore these themes in detail, highlighting how medical students perceive and experience failure.

In summary, this study’s methodological approach provided an in-depth understanding of medical students’ perspectives on failure. Conducting interviews via Microsoft Teams was efficient, and stringent data management procedures ensured compliance with ethical standards. The thematic analysis identified key patterns in the data, which will be discussed in further detail.

## Results

Key themes identified during the analysis

**Figure 4:**
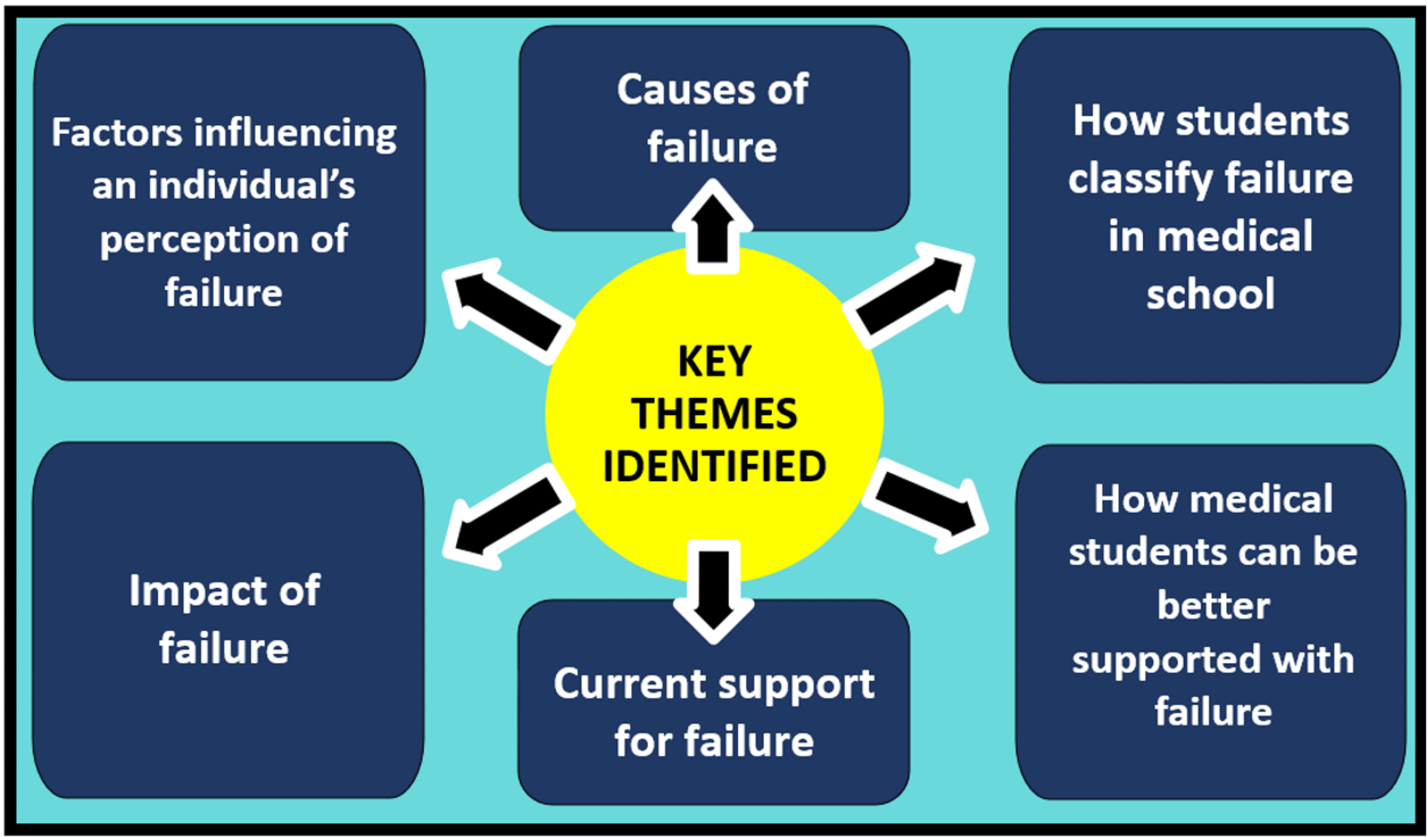
Diagram of key themes identified in the research study

Thematic analysis of the transcripts brought about six key themes, shown in Figure 4.

Theme one: factors influencing an individual’s perception of failure

Several factors influence a medical student’s perception of failure. All participants described failure initially as not meeting certain expectations or goals: “I would define failure. Uh. Umm. I guess when something doesn't go as expected,” (Student 2). The expectations from within could be a qualitative definition of the amount of effort they had put in for something: “..second and third year I would have seen failure as not trying,” (Student 5). Students acknowledged that sometimes they thought they had failed due to the personal internal expectations that they had set, being too high: “So, if I have my expectations way too high and I'm failing that, then possibly I need to lower my expectations,” (Student 6). Responses to failure were the greatest when attributed to educational impact such as not getting a foundation year job: “Also if I didn't get a job at all that would definitely have felt like a failure,” (Student 1).

External factors that affected medical students’ perceptions of failure were the medical school’s pass mark, clinicians, patients, family, friends, and peers. Medical school: “So, I was going on the medical school’s pass mark and so in my mind…I just needed to pass,” (Student 6). Clinicians: “the consultant used to make me feel really upset, I guess the reason why I felt upset is because he made me feel like I'm not meeting expectations of what a third year should know,” (Student 5). Patients: "there's now a sense of like, we can't fail our patients. And what we're learning now is about it's not just about the exam,” (Student 5).

Previous experiences of failure and transition points in education such as high school to medical school influenced perceptions of failure: “Before medical school uhh I think I probably looked negatively towards failure as it's just something where something goes wrong,” (Student 2). Perceptions of failure evolved and were the most significant at transition points: “then slowly by the time I came to Med school, my like mindset kind of shifted to… even if you pass this OK like because I think in Med school everyone is really smart,” (Student 3).

Perceptions of failure were complex with individuals having varying definitions: “I think that's when I realized that like failure means different things to different people," (Student 5). Some students perceive failure as all or nothing, especially when joining medical school. Progressing through medical school, failure evolves into a spectrum: “Yeah, I think it, I think for me it's a spectrum,” (Student 3).

Theme two: how students classify failure in medical school

Failure was classified into the following categories: something which stopped academic progression (not meeting the medical school pass mark) such as failing written assessments, not getting a foundation year job, presentations, observed structured clinical examinations (OSCEs), students selected components and essays. The second category was a failure in educational growth. Students described failure in this category as when there was a lack of enjoyment in learning, lack of productivity, not doing well in a clinical skill, not doing well on placement, lack of taking part in learning opportunities, or failing their patients. “The only placement really that I've felt successful throughout medical school was my GP placement this year because I felt for the first time useful. I felt like I was good at what I was doing, and I felt part of the team,” (Student 1). Unlike the first category where the description of failure was definitive and set by the medical school, with failure in educational growth the expectations were set from within the individual.

The last category of experiences of failure was personal or non-academic failure. “I can remember that one week strongly. It really was not a good time for me. Umm, but my family came to stay the week after that. Uh in London and it was strange because as soon as I was with them, like my mind, just like reset, it was just like fresh,” (Student 4). All participants prioritized the importance of academic failure over personal failure or non-academic failure. “I would say that what I personally think for me personally when I failed something in academia, I guess the impact of it mentally can last, and sometimes it can even last even after I've done well,” (Student 2).

Theme three: causes for failure

Causes for failure in medical school are complex and often multifactorial. The most common academic causes that students mentioned were poor study skills and attitudes toward studying. Lack of experience with failure and not knowing how to deal with failure caused further failure. This was due to bad coping habits in response to failures. Students lacked awareness of wider causes of failure and prioritized academic reasons for failure. This meant the default response was to work harder to cope with failure. “Sometimes you go into the spiral. You don't even revise…you would want the concept of failure to motivate you to study more, but then sometimes it becomes so consuming that you can't even sit and study,” (Student 3).

Psychological causes that were mentioned for failure were poor mental health, anxiety, and stress. Motivation was another concept that was heavily mentioned, often students who lacked motivation and struggled to study would end up failing exams. Some students mentioned the lack of self-efficacy and feelings of imposter syndrome after failure which then caused further failure. “I think like self-efficacy also comes into it there and if you don't believe that, you can do it, it makes it difficult to do it,” (Student 1).

Social reasons for failure included high parental expectations, poor working environment, and added responsibilities towards family. Due to changes in expectations and environment, transition points in education such as from pre-clinical to clinical years caused students to initially struggle to adapt and come to terms with the expectations. Those who struggled to adapt failed. Hesitancy to seek support and not seeking support on time contributed to failure. “They had a disability that would make it rather difficult for them to reach their expected goal. And so it would be more likely that they would fail… They weren't very open about it and they kind of brought it up to me like three weeks after knowing them and I was kind of like, dude, I could have helped you like three weeks ago,” (Student 6).

Some causes for failure are not preventable and instead, the goal or expectation needs to be changed. Students may find it difficult to seek reasonable adjustments if they have a physical or mental health condition or learning disability.“That's why reasonable adjustments exist as well for a disability because the expectation, the goal cannot physically be met,” (Student 6).

Theme four: impact of failure

Students mentioned that the biggest academic consequence of failure in medical school would be where it stops academic progression, within this category students mentioned getting a poor ranking, re-sitting exams, having marks capped, and hindering learning. Students in clinical years also had wider impacts such as poor patient outcomes: “We can't fail our patients. And what we're learning now is about it's not just about the exam, it's about real-life people,” (Student 5).

Students had negative coping mechanisms by withdrawing from social activities and were hesitant to seek support from students and faculty due to the fear of how people would view them which highlighted the stigma of failure: “They became more reserved…after they failed, second year and in terms of like outings and like going out like, yeah, like going out and having fun, they kind of restrict themselves in the way that they think….because I have failed, I kind of don't deserve to go out as much and have fun,” (Student 3).

Psychological impacts of failure led to a decrease in motivation in students, a reduction in confidence with reduced self-efficacy, and feelings of imposter syndrome. Students who did not cope with failure well had a lack of reflection after the failure feared failure, and failed to acknowledge what had happened. On the other hand, students who had positive coping mechanisms for failure dealt with failure well. They reflected on their experiences and used them as an opportunity to do better: “I think also it made me re-evaluate like where I was actually spending time… it built some more resilience, especially like when I did pass the second time…I think also because now that I have some sort of experience in failure I can give that to other people as well like whenever…they're going through a similar kind of situation” (Student 2). Most students when seeking support from peers would do so from individuals who had previously failed. What they learned from failure was better organization, consolidation of knowledge, and improved time management.

Theme five: support for failure

Students accessing formal modes of support such as student support, supervisors, and mentors had quite positive experiences. When mentors and supervisors holistically saw students and got students to reflect, this was the most beneficial. However, there are many barriers to accessing formal modes of support. Often students did not know what support was available and how to access it: “If you turn up to the wrong people, then they can't do anything about it. They can't help you. Then it can be quite a waste of energy. However, if you do go to the right people, I found that they could be really supportive and work out ways to help you reach the expected goal,” (Student 6).

Other students had misconceptions about accessing formal methods of support: "Again, I just thought like this support help thing would impact my degree as a whole,” (Student 3). Students felt like accessing formal modes of support were last resort options and accessing them would mean they were not doing well: ”I’ve always sort of done well academically and you know progressively I feel like if I'm someone that's getting help from students support then it means I'm not doing well or I must be you know seriously behind or something must be seriously wrong for me to be at a level where I actually need to request help from the medical school itself,” (Student 5).

Students also sought informal support from peers and student societies. There were barriers to seeking peer support as there was the fear of being judged for their failures since most individuals in medical school had not failed before. There was a negative stigma mentioned regarding failure which prevented people from being open about their failures: “So, knowing how like those people felt and knowing like they'd know how I'd feel, I felt comfortable going to them and speaking to them. I did eventually speak, reach out to maybe people who hadn't failed at all, and I think that was probably quite tough because not… they hadn't perhaps known what failure was in terms of academia and not knowing how they would react,” (Student 2). Student societies play a big hidden role in supporting students: “there seems to be this hidden support from societies…to like do revision sessions and help people out when they were struggling with like the content of the medical course,” (Student 6).

Theme six: how medical students can be better supported with failure

Multiple strategies for improving support for medical students were mentioned. Students stated that the medical school should improve signposting to medical school support to make it easier to access. The medical school should acknowledge and utilize informal methods of support from peers and societies. Support should be offered as a preventative measure instead of after failure: “…the fact that you only see senior tutors when you are failing, …I think that's a bit bad because they should be able to be there before you start failing. So, if you were to see them before like you got, say you got like 55% in the test or 57 or 60 or something like or you're on the cusp of failing and you don't see a senior tutor then, what's going to stop you from failing the next one, realistically?” (Student 6).

To effectively mentor students to cope with failure, participants mentioned it’s important to get students to reflect on their failures. Students will require help with acknowledgment and acceptance of failure. Once students fail, they need to reshape their expectations, which helps change their perceptions of failure. Breaking the stigma of failure is also important: and I think it's a pride thing. And also I think so you've cut off all of the people who are quite gifted academically, so you've got all these people who are very, very smart and so it makes you quite vulnerable to have to kind of go. Look, I failed this,” (Student 6).

Students suggested having more open discussions about failure, normalizing failing in medical school, and releasing statistics on how many people sought out help after struggling in medical school: “on the first day of medical school. If there's this one big slide that says. Make mistakes….I think that in itself sets the tone for the rest of the academic year…I think it's just normalizing it and I think that starts with faculty members normalizing it,” (Student 2).

## Discussion

Medical students' perceptions of failure are complex and multifactorial, as highlighted in previous literature [14]. Students define failure as "not meeting expectations" which varies based on individual tolerance levels. These expectations can be quantitative (e.g., exam percentages) or qualitative (e.g., study effort). A student may feel they have failed by scoring below 80%, even if the exam pass mark is 50% [15]. The internal consequences of failure are personal, as individuals choose their response as positive or negative. External factors, such as expectations from medical school, parents, peers, or society, influence students' views on failure. Adjustments like exam accommodations can affect whether a student meets expectations. Furthermore, external consequences, such as academic progression, impact how failure is perceived, with mental and emotional responses varying among students.

Students’ perceptions of failure evolve throughout medical school. Preclinical students often adopt an "all-or-nothing" approach. In contrast, clinical students view failure as a spectrum, better preparing them for the uncertainties of clinical practice, such as prescribing errors or patient outcomes [3]. This shift aligns with Knight’s research, which shows medical students move from a "black and white" view to a more nuanced understanding of failure [22]. Challenges arise when students cannot adapt to this change. Academic failure was prioritized over personal or educational growth, often leading to burnout during exam periods [6]. Clinical students, however, emphasized the importance of learning for patient care, recognizing that educational failure could harm patients.

Students often focus on academic causes of failure, neglecting underlying personal issues, which research shows are frequently the root causes [10]. Without addressing these, students may adopt ineffective coping strategies. Timely self-awareness and seeking support are crucial to preventing failure. Relying on medical schools to identify struggling students through tools, although effective, places a burden on institutions [23-36]. Teaching students to recognize their limits and seek support early is vital for both academic and clinical success. Students who coped well with failure used positive strategies, viewing failure as a learning opportunity to build resilience and develop a deeper approach to learning [4]. 

To improve support, students suggested integrating student societies with formal medical school support services, as seen in other studies [12, 14]. Breaking the stigma around failure requires open discussions, with faculty sharing their experiences or publishing statistics on student struggles. Enhancing visibility and access to resources would also encourage students to seek help.

## Conclusions

This study has highlighted the complexity of medical students’ perceptions of failure, exploring its causes, impact, and support mechanisms to provide a comprehensive understanding. Failure extends beyond academics, involving various factors and individuals that shape how students perceive it. As students progress through medical school, their view of failure evolves from a rigid 'all-or-nothing' mindset to a more nuanced, uncertain concept, similar to the nature of clinical medicine.

Understanding these perceptions is crucial to helping students adjust their expectations and prepare for the uncertainties of clinical practice. While failure has a significant impact, it can also lead to positive growth when managed well. Students often rely on informal support, such as peer networks, to cope. Integrating this informal support with formal resources would improve access and encourage students to seek help when needed. Normalizing discussions about failure in medical school, involving clinicians, hospital trusts, medical schools, patients, society, and students, would help reduce the stigma associated with it and foster a healthier approach to dealing with failure.

## Appendices

Appendix 1

Semi-structured interview questions used:

1. Please tell us a bit about yourself and your background.

2. What are your thoughts on the concept of failure?

3. How would you define failure?

4. What does the concept of failure mean to you in the context of medical school?

5. Do you see failure purely as an academic concept?

6. Have you had any experiences of failure?

7. How have you coped with failure? Could you reflect on this? If not, could give an example of a time you didn’t do as well as you expected?

8. How has failure affected you in medical school?

9. What do you think causes failure?

10. Have you ever sought out any help with dealing with failure? Who did you seek help from? Did you seek any help from the medical school? What were your experiences like?

11. How do you think students can be supported with the concept of failure?

12. Do you have any questions for me? Or anything else to add?
